# Atypical Presentation of Subcapsular Liver Hematoma With a Delayed Onset of Hemolysis, Elevated Liver Enzymes, Low Platelet Count (HELLP) Syndrome

**DOI:** 10.7759/cureus.74299

**Published:** 2024-11-23

**Authors:** Gargi Mukherjee, Naguib Fayez Naguib Guirgis,, Bini Ajay, Indranil Banerjee, Miriam Khalil

**Affiliations:** 1 Obstetrics and Gynaecology, Surrey and Sussex NHS Trust, Redhill, GBR; 2 Obstetrics and Gynaecology, Croydon University Hospital NHS Trust, London, GBR; 3 Obstetrics and Gynaecology, Oxford University Hospitals NHS Foundation Trust, London, GBR; 4 Radiology, Epsom and St Helier NHS Trust, London, GBR

**Keywords:** acute pain, hellp syndrome, liver disease in pregnancy, preeclampsia, subcapsular liver hematoma

## Abstract

Subcapsular liver haematoma in pregnancy, a rare and life-threatening condition, is more commonly associated with severe preeclampsia and haemolysis, elevated liver enzymes, and low platelet count (HELLP) syndrome. The common presenting symptom of subcapsular haematoma is acute-onset upper abdominal pain in patients suffering from preeclampsia; shock is the presenting feature in severe cases of rupture. Here we have discussed a case of subcapsular haematoma associated with HELLP syndrome in a patient who responded to conservative management. The diagnosis of subcapsular haematoma was delayed as the clinical presentation was atypical.

A 30-year-old primigravida with an apparently uncomplicated pregnancy attended with sudden onset acute abdominal pain in Croydon University Hospital, London, UK. Due to associated cardiotocography (CTG) concerns, the initial diagnosis was placental abruption. An urgent caesarean section was performed, and a spontaneous hemoperitoneum of 500 ml was found during the surgery. Following the caesarean section, the blood pressure started to rise. Subsequently, the blood picture deteriorated to full-blown HELLP syndrome, and the pain worsened. Further imaging revealed a large subcapsular haematoma of the liver. The patient was managed conservatively in the intensive therapy unit (ITU) by a multidisciplinary team including intensivists, an upper gastrointestinal surgeon, and an obstetric team. She responded to conservative management and had an uneventful recovery.

A high index of suspicion in cases of severe pain in the abdomen during pregnancy can help in early diagnosis. Moreover, further exploration in the case of spontaneous hemoperitoneum during caesarean section should be considered to avoid delay in diagnosis. Prompt intervention by the pertinent teams is the key to successful treatment.

## Introduction

Subcapsular haematoma of the liver during pregnancy is uncommon. Approximately 6% of all pregnancies are affected by preeclampsia; among all the patients with preeclampsia, around 9%-10% develop haemolysis, elevated liver enzymes, and low platelet count (HELLP)syndrome [1]. Only 1%-2% of all women with HELLP syndrome develop this rare complication of subcapsular haematoma of the liver [2].

Subcapsular haematoma develops in between Glisson's capsule and the liver parenchyma. The clinical feature includes non-specific severe upper abdominal pain sometimes radiating to the shoulder. The pain is very severe in nature and is not relieved by simple analgesics. In undiagnosed cases, the life-threatening complication is a rupture of the liver, which has significant mortality. The mortality in cases of subcapsular liver haematoma ranges from 17% to 59% [3].

Here we discuss a case report of a 30-year-old pregnant woman who presented with acute-onset pain in the abdomen. She was initially diagnosed with concealed placental abruption; later, imaging diagnosed a subcapsular haematoma of the liver, which was managed conservatively by the multidisciplinary team.

## Case presentation

A 30-year-old Caucasian lady, P0, low risk at booking, presented to maternity triage with the complaint of acute onset of abdominal pain at 30+5 weeks of gestation in Croydon University Hospital, London, UK. She had attended antenatal appointments regularly, and pregnancy was uncomplicated so far. She had regular antenatal visits. Her blood pressure (BP), urine check, fundal height measurement, 11-13-week scan, and anomaly scan, all findings were within normal limits. Her pregnancy was considered low-risk so far.

She complained of sudden onset of pain in the abdomen and back radiating to the shoulder for the last 10 hours. The pain was persistent, sharp, and stabbing and not relieved by regular pain relief. It was not associated with any nausea, vomiting, urinary symptoms, or bleeding per vagina.

The observation showed a pulse rate of 102 beats/minute, BP of 136/86 mm of Hg, respiratory rate of 20 breaths/minute, and normal oxygen saturation. There was the presence of 1+ of protein (mild proteinuria) in the urinalysis.

On clinical examination, generalised tenderness in the abdomen, both upper and lower, was noted but no peritonism. The uterus was tender and toned up. On speculum examination, the cervical os was closed with no bleeding.

Cardiotocography (CTG) showed a baseline of 165, reduced variability, and spontaneous unprovoked decelerations. In view of the sudden onset of abdominal pain and abnormal CTG findings, the provisional diagnosis was concealed placental abruption. The absence of raised blood pressure and other preeclampsia symptoms (headache, visual disturbance) at the presentation did not raise the suspicion of a complication associated with preeclampsia. Additionally, the CTG abnormalities suggested more abruption. Finally, the patient presented late at night, and the absence of a senior consultant led the junior doctors to overlook the offbeat and rare condition of such acute pain. 

A category 1 caesarean section was performed. The blood results before and six hours after the procedure are presented in Table 1.

**Table 1:** The patient's blood results before and after delivery

Parameters	In triage (before delivery)	Six hours post delivery	24 hours post delivery
Hemoglobin (g/L)	98 g/L	74 g/L	100 g/L
Platelet	167 x 10^9/L	126x10^9/L	64X 10^9/L
White cell count (WCC)	7.8x10^9/L	7.6x 10^9/L	8.6 x 10^9/L
Creatinine	30 umol/L	35 umol/L	32 umol/L
Alanine transaminase (ALT)	29 U/L	146 U/L	656 U/L
Protein-creatinine ratio	-	123 mg/mmol	187 mg/mmol

During the caesarean section, a spontaneous hemoperitoneum of 500 ml was noted. A non-vigorous live foetus was delivered; however, no significant retroplacental clot was identified. It was thought that the abruption had caused a backflow of blood through the fallopian tube, causing the hemoperitoneum. The abdomen was closed with a drain. (Total blood loss: -1.5 litres). Since she has lost 1.5 litres, the routine major obstetric haemorrhage protocol (RCOG) was initiated. However the intraoperative venous gas showed haemoglobin of 88 g/L, and no blood was transfused intraoperatively. The BP in the recovery period started to rise 150-160/100-110 mm of Hg and was commenced on labetalol 200 three times a day. High BP and blood picture of low haemoglobin, reduced platelets, and raised liver enzymes indicated HELLP syndrome. The patient was closely monitored, including hourly observation, hourly urine output, and six hourly blood. She also received transfusions of four units of red blood cells and four units of fresh frozen plasma (FFP) postoperatively. The haemoglobin after transfusion was 100 g/L and remained static, but the platelets were persistently dropping, reaching 64X 10^9/L, and alanine aminotransferase (ALT) increased to 656 U/L at 24 hours post delivery. The other parameters, like coagulation screen, lactate dehydrogenase (LDH), and renal function, remained stable throughout. She was haemodynamically stable with normal urine output. The collection in the drain remained minimal with blood-stained serous discharge.

**Figure 1:**
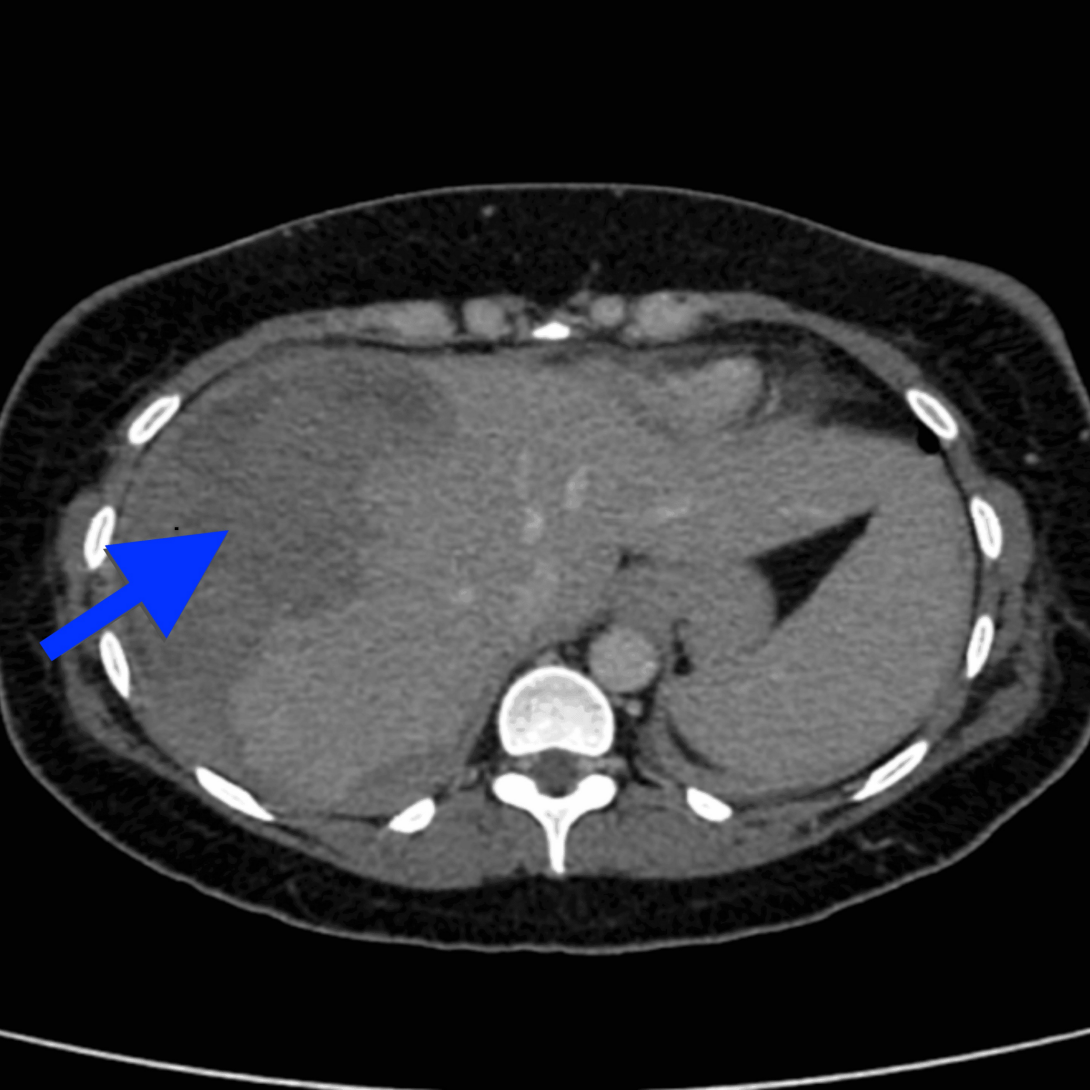
Axial section of the CT scan performed on day one showing a large subcapsular hepatic haematoma

**Figure 2:**
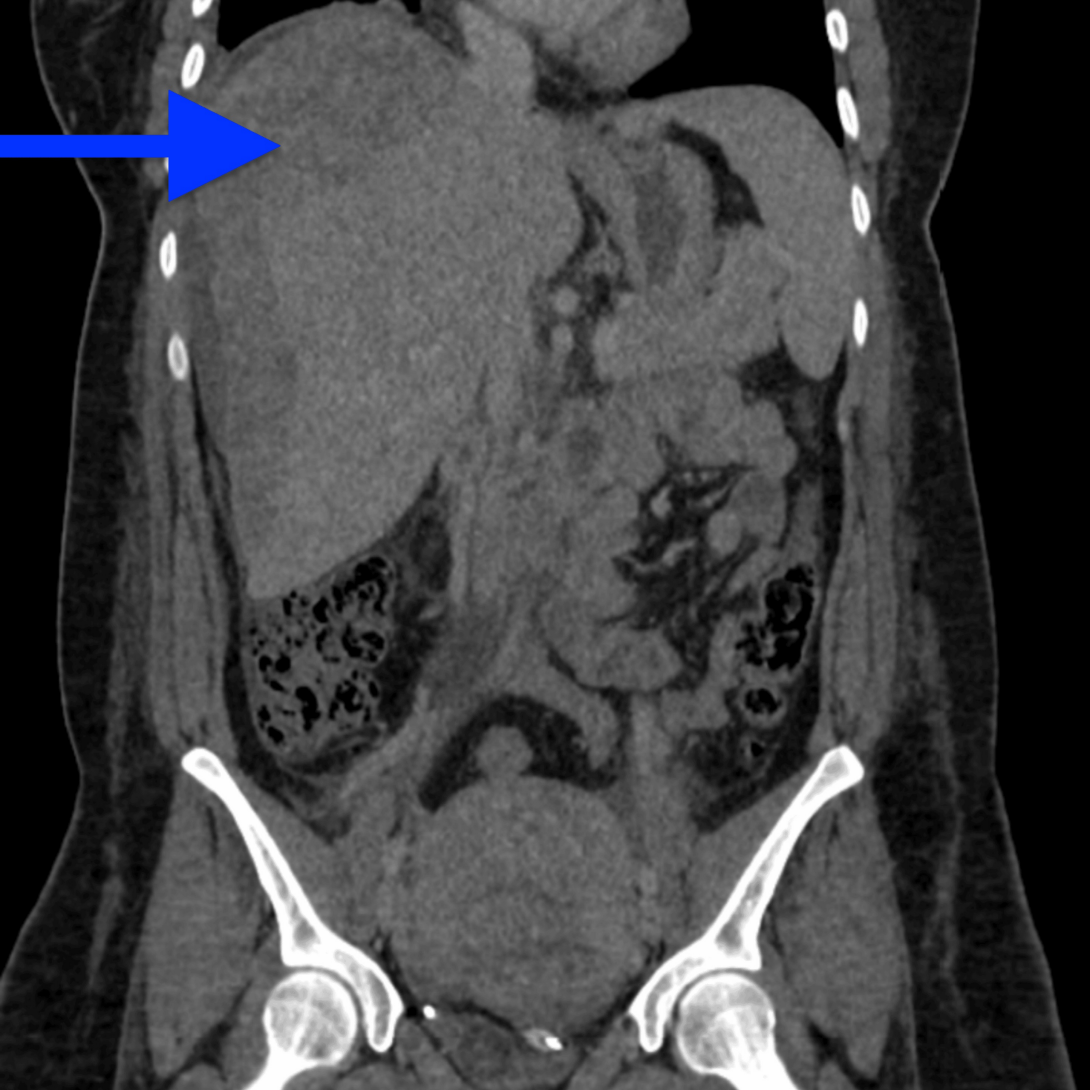
Coronal view of the CT performed on day one, showing a large subcapsular hepatic haematoma (blue arrow)

However, the pain in the abdomen did not improve even after 24 hours of surgery, and it became localised mostly in the upper abdomen, radiating to the back. An urgent CT scan of the whole abdomen was done, which showed a large subcapsular haematoma of 15x8x9 cm adjacent to the right hepatic lobe (Figures 1, 2).

Although the observations were overall stable except for slight tachycardia (96-104/min), considering the worsening blood picture and large subcapsular liver haematoma, she was transferred to ITU in the tertiary care unit. In the intensive therapy unit (ITU), she was managed conservatively by the joint team of ITU, hepatobiliary surgeons, and obstetricians. Her BP remained stable at 130-140/80-95 mm of Hg on regular amlodipine 10 mg. She received another two units of blood and received a course of intravenous antibiotics.

**Figure 3:**
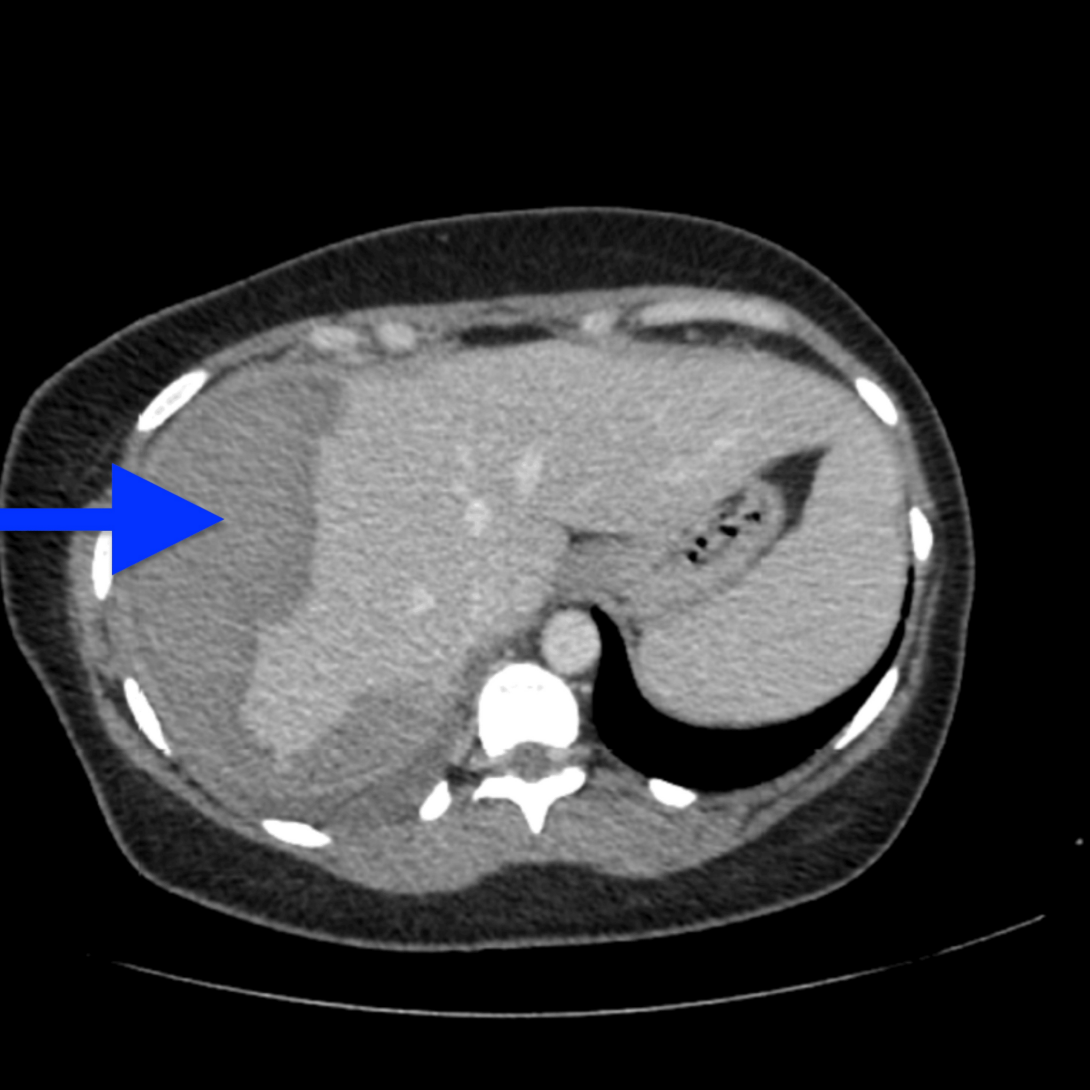
Axial section reformate through the upper abdomen on day 15 showing a stable subcapsular hepatic haematoma (blue arrow)

**Figure 4:**
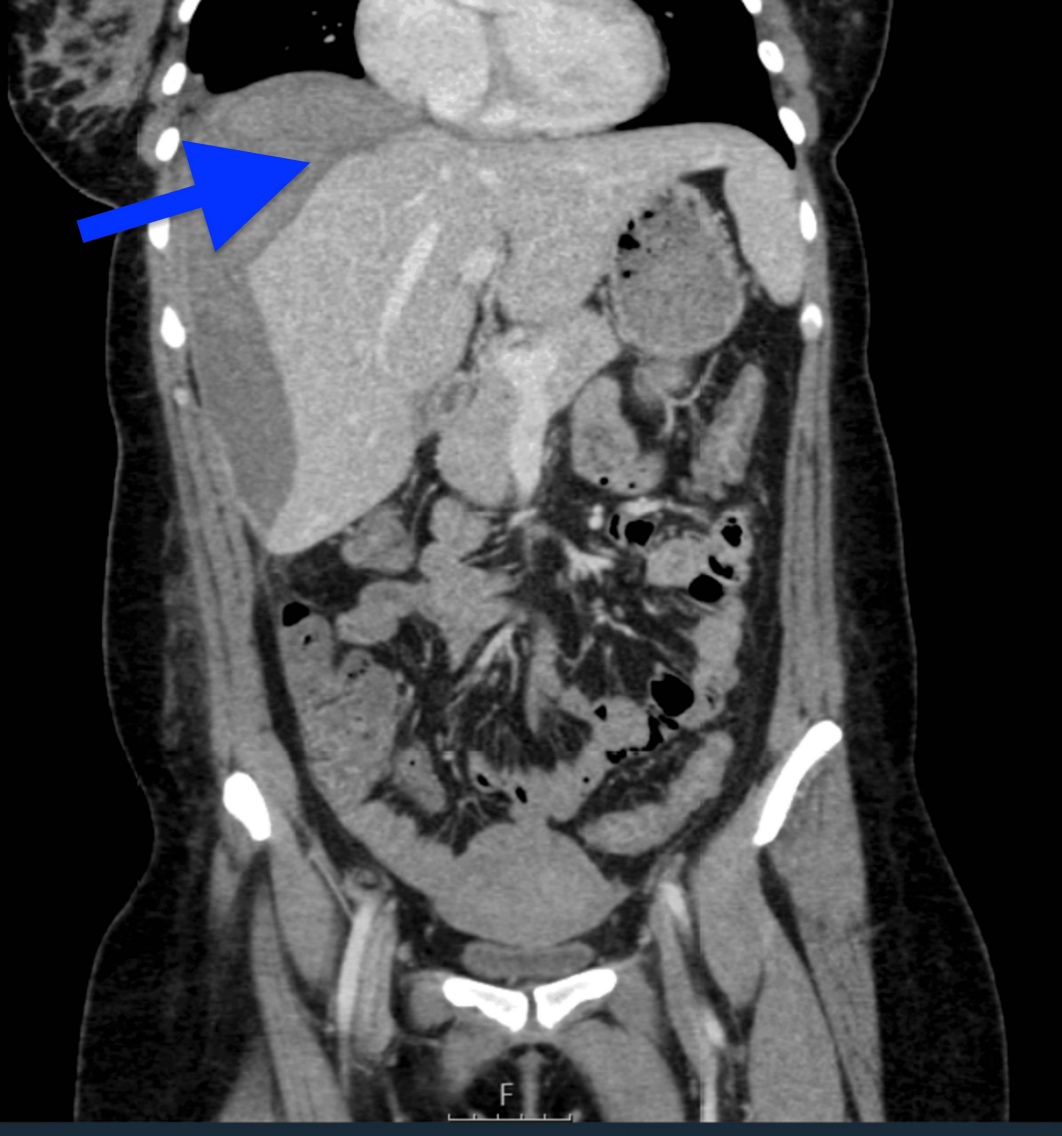
Coronal section reformate through the upper abdomen on day 15 showing a stable subcapsular hepatic haematoma (blue arrow)

A repeat CT scan on postoperative day five showed no evidence of an increase in the size of the haematoma or active bleeding. The blood reports remained stable, and at the end of day five haemoglobin was 94 g/L, platelet count was 405x10^9/L, and ALT was 46 U/L. The rest of the blood parameters, like renal function test, LDH, and coagulation screen, were stable throughout. The repeat CT scan on postoperative day 15 (Figures 3, 4) also showed the haematoma was stable with no increase in size. The patient was stable with minimal pain in the abdomen (not requiring any pain relief), normal BP, and normal blood parameters. She was discharged on day 20 with the follow-up plan of a monthly ultrasound scan. The follow-up ultrasound scan in the successive three months showed a reduction in the size of the haematoma to 5x5x6 cm.

## Discussion

Spontaneous subcapsular liver haematoma and liver rupture is a rare and potentially life-threatening situation. It was first described by Abercombie in 1844 [4]. The incidence is approximately one in 45,000 to 2,25,000. It is more commonly associated with preeclampsia, specifically HELLP syndrome. Approximately 1%-2% of patients with HELLP syndrome develop sub-capsular hematoma. Other risk factors include multiparity and age greater than 30 years [5].

The etiopathology is still unclear and there are multiple hypotheses. It can be due to microangiopathy causing liver ischaemia, activation of complement, microthrombosis, and endolytic injury causing hepatic sinusoidal obstruction [2,6]. The associated neovascularisation of the hepatic parenchyma tends to bleed more in hypertensive episodes, leading to the formation of haematoma. This haematoma stretches the Glissen’s capsule and, in more severe conditions, ruptures. The most common site of haematoma is on the right side [7].

The common signs and symptoms include epigastric pain (70%) and hypertension (65%). The pain is usually severe in the upper quadrant and epigastrium, sometimes radiating to the shoulders, and not resolved by regular pain relief. Patients with liver rupture presented with symptoms of shock [8].

In the aforementioned case, the absence of hypertension and the presence of associated CTG abnormalities led to the difficulty in diagnosis. Considering the rarity of the incidence of spontaneous subcapsular haematoma, the more common condition like concealed placental abruption was the working diagnosis. Moreover, the normal BP and blood results at presentation also hindered the diagnosis. This is a relatively rare variant where the clinical picture of hypertension and HELLP syndrome emerged following the development of liver haematoma.

Ultrasound scan is usually the first line of investigation [9,10]. The alternatives include a CT scan or MRI; a CT scan with contrast is particularly beneficial to diagnose active bleeding. Since the patient was investigated postpartum, CT was an acceptable alternative.

The management of patients depends on the severity of the symptoms. An exploratory laparotomy should be considered in deteriorating patients and cases of suspected rupture [11]. This allows confirmation of diagnosis and treatment along with delivery of the baby. The acceptable treatment includes digital compression of the hepatic artery to arrest bleeding temporarily, evacuate the haematoma and temporary packing with large gauze swabs. Once the patient is stabilised, a repeat laparotomy is performed to remove the gauze [12].

Unruptured haematoma is usually managed conservatively with serial imaging. However, if there is evidence of an increase in the size of haematoma in the serial imaging or there is deterioration of the clinical condition, surgical management should be considered [13]. Delayed ruptures of up to six weeks have been reported; regular, cautious follow-up is warranted in the cases [14]. Hepatic embolisation is an alternative method in selected units where the facility is available [14,15]. The risk includes ischaemic necrosis of the liver, hepatic failure, sepsis, and hepatic rupture [8].

The presence of a hemoperitoneum during a caesarean section could have warranted further exploration during the surgery. It is indeed recommended to explore further the reason for unexplained hemoperitoneum in the absence of uterine pathology. However, since the provisional diagnosis was a concealed abruption, the hemoperitoneum was accounted for due to the backflow of blood from the fallopian tubes. An additional intra-abdominal drain was inserted for subsequent monitoring. Further, since the patient presented in unsocial hours, additional input from the senior member of the team was not available.

However, in the postoperative period, since the condition did not improve, further investigation was initiated promptly. Following the diagnosis of subcapsular haematoma, a multidisciplinary team discussion with the upper gastrointestinal team and ITU team was done, and the patient was transferred to the ITU for intensive monitoring. Since she remained haemodynamically stable, her regular blood did not show any worsening; she was managed conservatively, and her follow-up plan was arranged. 

Such critical cases should always be managed by a multidisciplinary team [16]. She had stayed haemodynamically stable, so conservative management with serial imaging was a suitable management option [17]. Since there is a record of delayed rupture [8], the patient was safety-netted accordingly. If there was any episode of pain in the abdomen or a fainting attack, she was advised to attend the emergency department immediately; regular follow-up scans and clinic appointments were booked. Although the presentation was atypical, the case was diagnosed and managed well. 

## Conclusions

Subcapsular haematoma of the liver is a rare and potentially life-threatening condition. The presentation can be variable. The extreme end of the spectrum is a hypovolemic shock due to the rupture of hematoma. The treatment of the condition depends on the clinical picture. The haemodynamically stable patient with stable blood should be managed conservatively by the multidisciplinary team in a tertiary care centre. The haemodynamically unstable cases or progressively deteriorating patients are treated surgically. To summarise, a high index of suspicion, early diagnosis, prompt intervention, and a multidisciplinary approach to treatment are key to the successful management of this rare and potentially lethal condition.
